# External magnetic field promotes homing of magnetized stem cells following subcutaneous injection

**DOI:** 10.1186/s12860-017-0140-1

**Published:** 2017-05-26

**Authors:** Yu Meng, Changzhen Shi, Bo Hu, Jian Gong, Xing Zhong, Xueyin Lin, Xinju Zhang, Jun Liu, Cong Liu, Hao Xu

**Affiliations:** 1 Department of Nephrology, the First Hospital Affiliated to Jinan University, No. 613 Huangpu West Road, Guangzhou, 510630 China; 2 Department of Radiology, the First Hospital Affiliated to Jinan University, No. 613 Huangpu West Road, Guangzhou, 510630 China; 3 Department of Nuclear Medicine, the First Hospital Affiliated to Jinan University, No. 613 Huangpu West Road, Guangzhou, 510630 China; 4 Shenzhen Engineering Laboratory for Genomics-Assisted Animal Breeding, BGI-Shenzhen, Shenzhen, 518083 China

**Keywords:** Mesenchymal stem cells (MSCs), Stem cells, UC, Magnetic resonance imaging (MRI), Rat

## Abstract

**Background:**

Mesenchymal stem cells (MSCs) are multipotent stromal cells that have the ability to self-renew and migrate to sites of pathology. In vivo tracking of MSCs provides insights into both, the underlying mechanisms of MSC transformation and their potential as gene delivery vehicles. The aim of our study was to assess the ability of superparamagnetic iron oxide nanoparticles (SPIONs)-labeled Wharton’s Jelly of the human umbilical cord-derived MSCs (WJ-MSCs) to carry the green fluorescent protein (GFP) gene to cutaneous injury sites in a murine model.

**Methods:**

WJ-MSCs were isolated from a fresh umbilical cord and were genetically transformed to carry the GFP gene using lentiviral vectors with magnetically labeled SPIONs. The SPIONs/GFP-positive WJ-MSCs expressed multipotent cell markers and demonstrated the potential for osteogenic and adipogenic differentiation. Fifteen skin-injured mice were divided into three groups. Group I was treated with WJ-MSCs, group II with SPIONs/GFP-positive WJ-MSCs, and group III with SPIONs/GFP-positive WJ-MSCs exposed to an external magnetic field (EMF). Magnetic resonance imaging and optical molecular imaging were performed, and images were acquired 1, 2, and 7 days after cell injection.

**Results:**

The results showed that GFP could be intensively detected around the wound in vivo 24 h after the cells were injected. Furthermore, we observed an accumulation of WJ-MSCs at the wound site, and EMF exposure increased the speed of cell transport. In conclusion, our study demonstrated that SPIONs/GFP function as cellular probes for monitoring in vivo migration and homing of WJ-MSCs. Moreover, exposure to an EMF can increase the transportation efficiency of SPIONs-labeled WJ-MSCs in vivo.

**Conclusions:**

Our findings could lead to the development of a gene carrier system for the treatment of diseases.

**Electronic supplementary material:**

The online version of this article (doi:10.1186/s12860-017-0140-1) contains supplementary material, which is available to authorized users.

## Background

There is significant potential for the use of mesenchymal stem cells (MSCs) in cell therapy [1]. However, their clinical application still faces various challenges, such as the fact that an efficient strategy for stem cell homing to target sites has not yet been identified. Several other factors limit the clinical application of stem cells, including the time and method of drug administration, cell concentration, the transmission medium, and cell homing [1, 2]. Homing of stem cells is achieved through direct local injection, local perfusion, and systematic administration. However, intravenous injection of stem cells results in the accumulation of a significant number of cells in the lungs and spleen, with a very low percentage of cells reaching the arterial system (about 5%), and an even lower percentage reaching the target organ (0.0005%) [2, 3]. Simple direct injections and local organ perfusions are limited to superficial organs or to organs directly connected to the main artery. In fact, although an intra-arterial injection ensures highest cell numbers for transplant, this method of stem cell transplantation increases the death rate by 41%, with animals succumbing to arterial embolism [4]. Therefore, an important prerequisite for treatments is to transplant MSCs with a differentiation potential directly to the target area.

Compared with traditional preparations, magnetic targeted drug delivery systems, which have been studied for years [5–8], are characterized as methods for improving drug targeting, enhancing the curative effect of drugs, and decreasing toxic side effects. Superparamagnetic iron oxide nanoparticles (SPIONs) are an excellent transmission medium based on the magnetic targeted drug delivery system [9–14]. Recent studies have revealed that labeling stem cells with magnetic nanoparticles for magnetic resonance imaging (MRI)-mediated tracking of stem cells has evolved. An improved curative effect on common carotid artery injuries was observed using magnetized endothelial progenitor cells, obtained from in situ intra-arterial treatment of spinal cord injured animals, using a magnetic field to direct the stem cells [15]. Recently, studies have used anionic magnetic nanoparticles to load endothelial progenitor cells, and have successfully controlled cell movement in the vessel network using a magnetic field [16]. Although the results are exciting, most of these studies involved the use of a constant electromagnetic field or an internal magnetic field.

Studies involving noninvasive external magnetic fields (EMFs) with the use of permanent magnets are rare. The superparamagnetism of SPIONs can be utilized to bring about directional movement of magnetized stem cells under the influence of an EMF. In the present study, human umbilical cord MSCs were transfected with SPIONs and green fluorescent protein (GFP), and injected into the subcutaneous tissues of nude mice, specifically into partial cells at some cell intervals, following a skin injury. In summary, although some studies have shown that noninvasive EMFs can increase magnetized cell homing following an intra-arterial injection, intravenous injection, or intrathecal injection, no evidence has been provided showing the same effect following a subcutaneous injection. The current study tested the hypothesis that an EMF can promote homing and guide the magnetized stem cells to make rapid directional movements, following subcutaneous injection. MRI as well as fluorescence imaging was used to track the stem cells in vivo. Our findings demonstrated an improved method of injury treatment, using MSCs as drug or gene carriers, which can also be applied for directional drug treatment or gene therapy, both of which have a significant importance in the clinical setting.

## Methods

### Cell culture

The study was approved by the regional medical ethical review board (Jinan University and Shenzhen people’s hospital). After obtaining a written informed consent, the human umbilical cord Wharton’s Jelly-derived MSCs (WJ-MSCs) were isolated as described previously [15], from the umbilical cords of full-term newborns, delivered at the Shenzhen People’s Hospital, Guangdong, China. Cells were cultured in low glucose-DMEM (Hyclone, Logan, Utah, USA) containing 2 ng/mL of basic fibroblast growth factor (bFGF) and 10% fetal bovine serum (FBS) (Gibco, Gran Island, NY, USA), and maintained at 37 °C in a humidified atmosphere of 5% CO_2_. The medium was replaced every 3 days, and the human umbilical cord-derived MSCs (HUCMSCs) were collected by trypsin (0.25%, Invitrogen, USA) digestion. All experiments were performed using MSCs at 3–5 passages [17].

### Flow cytometry analysis

Passage 3 WJ-MSCs were trypsinized, dissociated into a single cell suspension, and allowed to reach 60% confluency. Cells were then rinsed with phosphate buffered saline (PBS) and incubated with anti-human CD73-PE (BioLegend, 344,004), anti-human CD105-PE (BioLegend, 323,206), anti-human CD90-PE (BioLegend, 328,110), anti-human CD34-PE (BioLegend, 343,506), and anti-human CD45-FITC (BioLegend, 304,006) for 15 min at room temperature. After incubation, the cells were rinsed with PBS, read on a FACSCalibur (BD, USA) flow cytometer, and analyzed using the WinMDI 2.8 software. Mouse IgG1-PE (BioLegend, 400,114) and mouse IgG1-FITC (BioLegend, 400,107) were used as the isotype controls.

### Osteogenic differentiation

At approximately 80% confluency, WJ-MSCs were rinsed with PBS and cultured in osteogenic differentiation medium (Gibco, Gran Island, NY, USA). The medium was changed twice a week, and after 3 weeks, the cells were washed with PBS and fixed in 4% paraformaldehyde for 30 min. The cells were then stained with 0.1% Alizarin Red S water solution for 30 min.

### Adipogenic differentiation

At 80% confluency, WJ-MSCs were rinsed with PBS and cultured in adipogenic differentiation medium (Gibco, Gran Island, NY, USA). The medium was changed twice a week, and after 3 weeks, the cells were washed with PBS and fixed in 4% paraformaldehyde for 30 min. The cells were then stained with 0.3% Oil Red O solution for 30 min.

### Lentiviral transfection of WJ-MSCs

A lentiviral vector (Fitgene, Guangzhou, China) expressing GFP with a cis-acting element, CMV-GFP-puro, was packaged and used to infect the WJ-MSCs, according to the manufacturer’s protocol. The transduced cells were grown in low glucose-DMEM containing 2 ng/mL bFGF, 10% FBS, and 400 μg/mL puromycin. Puromycin-resistant WJ-MSCs, overexpressing GFP, were obtained after 3 days of puromycin selection. Stable clones were identified by the expression of GFP protein [16]. The efficiency of the target cells was estimated by fluorescence microscopy using an inverted fluorescence microscope--the GFP positive cells in each field (10X) visible/all the cells in each field (10X).

### Labeling of WJ-MSCs with SPIONs

Approximately 1 × 10^3^ genetically modified WJ-MSCs were seeded into each well of a 24-well plate. After 12 h of incubation in MSC growth medium, the cells were magically labeled with SPIONs (25 μg/mL; SPIONs-MSCs) (Molday ION Rhodamine B™, BioPhysics Assay Laboratory, Inc., Worcester, MA, USA; CL-50Q02-6A-50) and complexed to poly-L-Lysine (0.75–1 μg/mL) (BioPhysics Assay Laboratory, Inc., CL-00-01). The cells were then collected, washed twice in PBS, counted, and resuspended at the appropriate cell density for in vivo analyses.

### Cell vitality test

For Trypan blue staining, 200 μL of cells was aseptically transferred to a 1.5 mL clear Eppendorf tube, and incubated for 3 min at room temperature with an equal volume of 0.4% (*w*/*v*) Trypan blue solution prepared in 0.81% NaCl and 0.06% (*w*/*v*) dibasic potassium phosphate. Cells were counted using a dual-chamber hemocytometer and a light microscope. Viable and nonviable cells were recorded separately, and the means of the two cell counts were pooled for analysis.

### Full-thickness skin defect model and cell transplantation

In total, 30 specific pathogen-free BALB/C nude mice were randomly divided into 3 groups: 10 mice in the control group, 10 mice in the group receiving SPIONs-MSCs, and 10 mice in the group receiving SPIONs-MSCs exposed to an EMF (SPIONs-MSCs + magnetic field). The full-thickness skin defect model was implemented in all experimental mice, and cell transplantation was performed at a 1.5 cm distance from the wound using a 1 mL syringe. The control group was injected with 200 μL 0.9% normal saline, the SPIONs-MSCs group was injected with 2 × 10^6^ SPIONs and GFP double-labeled MSCs, and the SPIONs-MSCs + magnetic field group was injected with 2 × 10^6^ SPIONs and GFP double-labeled MSCs exposed to an EMF generated by a permanent magnet for 6 h/day. The effects of different EMF exposure times on stem cells were analyzed by the MTT assay and western blotting, by determining the apoptosis markers (Additional file 1: Figure S1). The full-thickness skin defect model was generated as follows: mice were anesthetized by an intraperitoneal injection of 10% chloral hydrate (0.3 mL/100 g); next, following disinfection, a 4-mm diameter circular skin defect was created to the depth of the deep fascia on the back of the mice, near the double hind limb. After completion of stem cell transplantation, a representative animal from each group was immediately subjected to MRI and in vivo fluorescence examination for baseline analysis. Three mice per group were subjected to MRI and fluorescence imaging at 24 h, 48 h, and 7 days post-cell transplantation, and the healed skin lesions were removed for pathological examination on day 7. Healed wounds were identified by the presence of the following: wound closure, epithelium, proper activity intensity, no wound dehiscence, no ulceration, an appropriate time lapse after wound generation, the ability to tolerate a certain tension and pressure at the wound site, gradual fading of skin color at the wound site, and similarity of the wound skin color to that of the surrounding healthy skin to maintain the skin barrier integrity. All experimental procedures were approved by the Animal Experimentation Ethics Committee of First Hospital Affiliated to Jinan University, Guangzhou, China.

### In vivo injection of magnetic WJ-MSCs

Mice were anesthetized with isoflurane (4% induction, 1.5% maintenance). The wounds were exposed to a magnetic field of 0.5 T using a small permanent neodymium (FeNdB) magnet (8 × 2 mm) for 6 h/day (Additional file 2: Figure S2). Subsequently, 2 × 10^6^ WJ-MSCs, which were previously magnetized using 25 μg/mL of SPIONs, were hypodermically injected (hypodermis) with 150 μL of PBS. Control animals received an identical cell infusion without the magnet implantation. Magnets were placed for 6 h daily.

### In vivo MRI

The animals used were BALB/C (nu/nu) nude mice (*n* = 3). One day, two days, or seven days after transplantation, the mice were anesthetized using chloral hydrate and underwent in vivo MRI (gradient echo scan), using a 3 T MRI scanner (Discovery MR750, GE Healthcare, USA). For image acquisition, a quadrature birdcage volume coil of 7 cm inner diameter was used. Axial images were taken with the following parameters: field of view = 4 × 4 cm^2^, matrix size = 256 × 256, slice thickness = 1 mm, TE = 6 ms, and TR = 700 ms. Following completion of the scan, the raw data and images were processed using the built-in professional software Discovery MR750, with each injected area defined as a unit, and using each of the 4 selected regions of interest (ROI) for measurements. MRI parameters included injection of cell volume, displacement, carrier-to-noise ratio (CNR), and signal-to-noise ratio (SNR).

### Fluorescence stereomicroscopy

To assess the distribution of SPIONS-MSCs in vivo, anesthetized rats were imaged for GFP fluorescence using a whole-body imaging system (IVIS Lumina II, Caliper, France). Filters of 480 nm (±10 nm) and 505 nm (±5 nm) represented the excitation and emission signals, respectively. High-resolution images were captured directly on a computer and analyzed using Living Image software (Xenogen Corporation, Almeda, California, USA). Results were expressed as number of photons/s/ROI.

### Prussian blue staining and tissue specimens

Prussian blue staining was performed to identify the SPIONs-MSCs. Cells were incubated for 30 min with 2% potassium ferrocyanide in 6% hydrochloric acid, and then counterstained with nuclear fast red for 30 s. A blue color indicated the presence of iron within the cells, thereby corresponding to the SPIONs-MSCs. Similarly, in skin tissue sections, SPIONs appeared as blue precipitates in the cytoplasm and pink in the nucleus. Tissue specimens were frozen in optimal cutting temperature (OCT) compound (Sakura Finetek Inc., Torrance, California, USA) in liquid nitrogen, and 10-μm sections were prepared using a cryostat microtome (CM1850; Leica Microsystems GmbH).

### Statistical analysis

The statistical significance of intergroup differences was assessed using the Student’s *t*-test or ANOVA followed by Tukey’s post hoc test. A *P* value <0.05 was considered statistically significant at the 95% confidence level. All values in the bar and line graphs are expressed as mean ± standard deviation (SD). The number of independent experiments analyzed has been stated in each figure legend.

## Results

### Lentivirus infection and SPIONs labeling

**Fig. 1:**
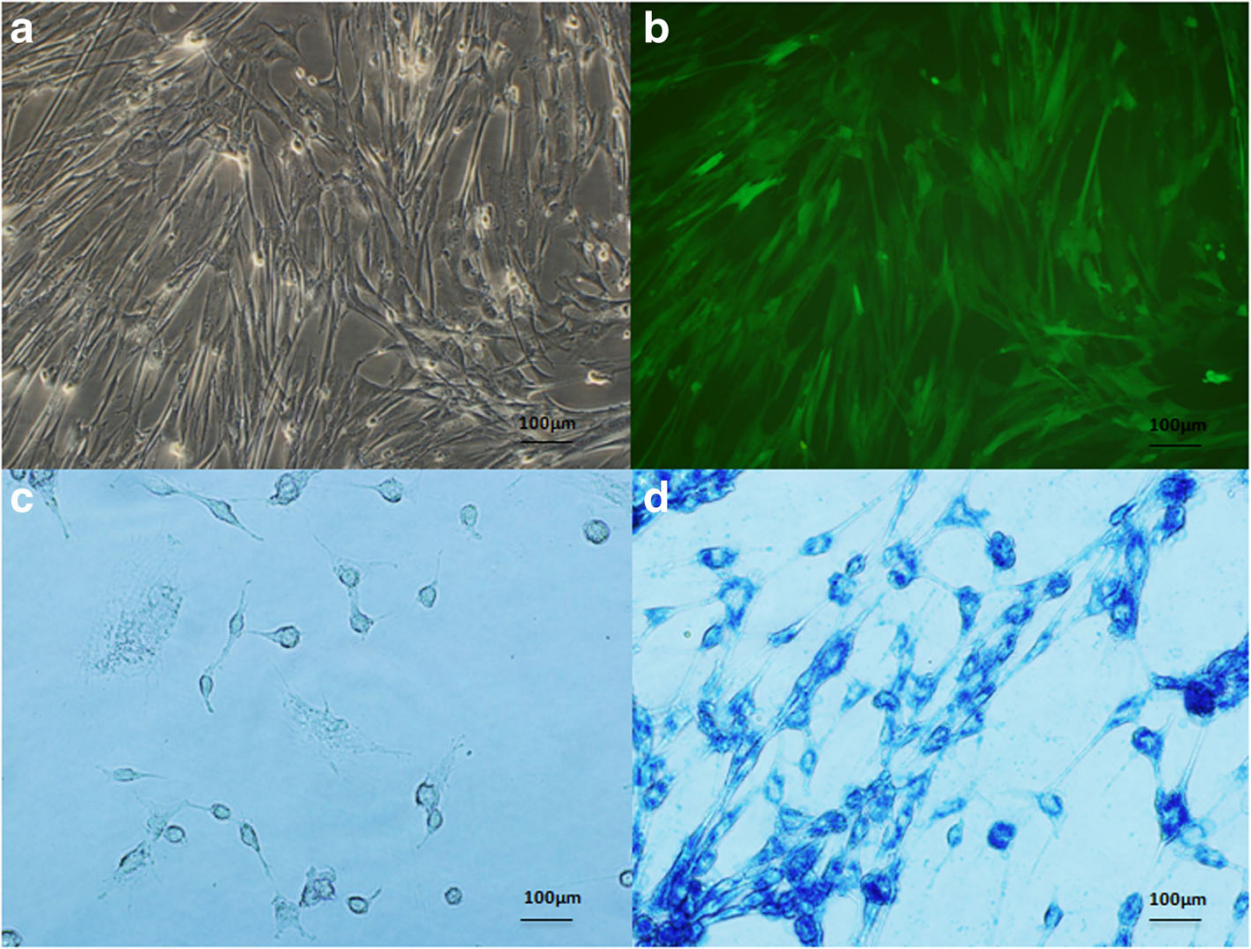
WJ-MSCs labeled with GFP/SPIONs. GFP-positive cells under fluorescence microscope (**a**) and (**b**). Cells labeled with SPIONs (**c**) and (**d**)

In our study, GFP was used for the in vivo tracking of WJ-MSCs. GFP was incorporated into a lentiviral vector containing independent puromycin expression frames. WJ-MSCs were isolated from fresh umbilical cords and cultured in MSC medium for several passages. Lentivirus-infected WJ-MSCs were selected in MSC medium with puromycin for 3 days. Stable clones were GFP positive (>99%), as detected by fluorescence microscopy (Fig. 1a and b). GFP expression was observed under a fluorescence microscope. Using an inverted fluorescence microscope**,** we observed the HUCMSCs for a green fluorescence signal at 12 h post-transfection; however, the signal was weak and expressed only by a few cells. The number of GFP-positive cells increased constantly 24 h post-transfection, with 4-10 GFP-positive cells in each visual field (10X) at 48 h, and more than 10 GFP-positive cells in each visual field (10X) at 72 h. The GFP transfection efficiency with lentivirus infection was over 99%. The GFP-positive WJ-MSCs were then transfected with SPIONs, and the transfection efficiency was evaluated by Prussian blue staining. Results demonstrated that more than 80% of the cells were labeled with SPIONs (Fig. 1c and d).

### Immunophenotype, differentiation potential, and vitality of WJ-MSCs

**Fig. 2:**
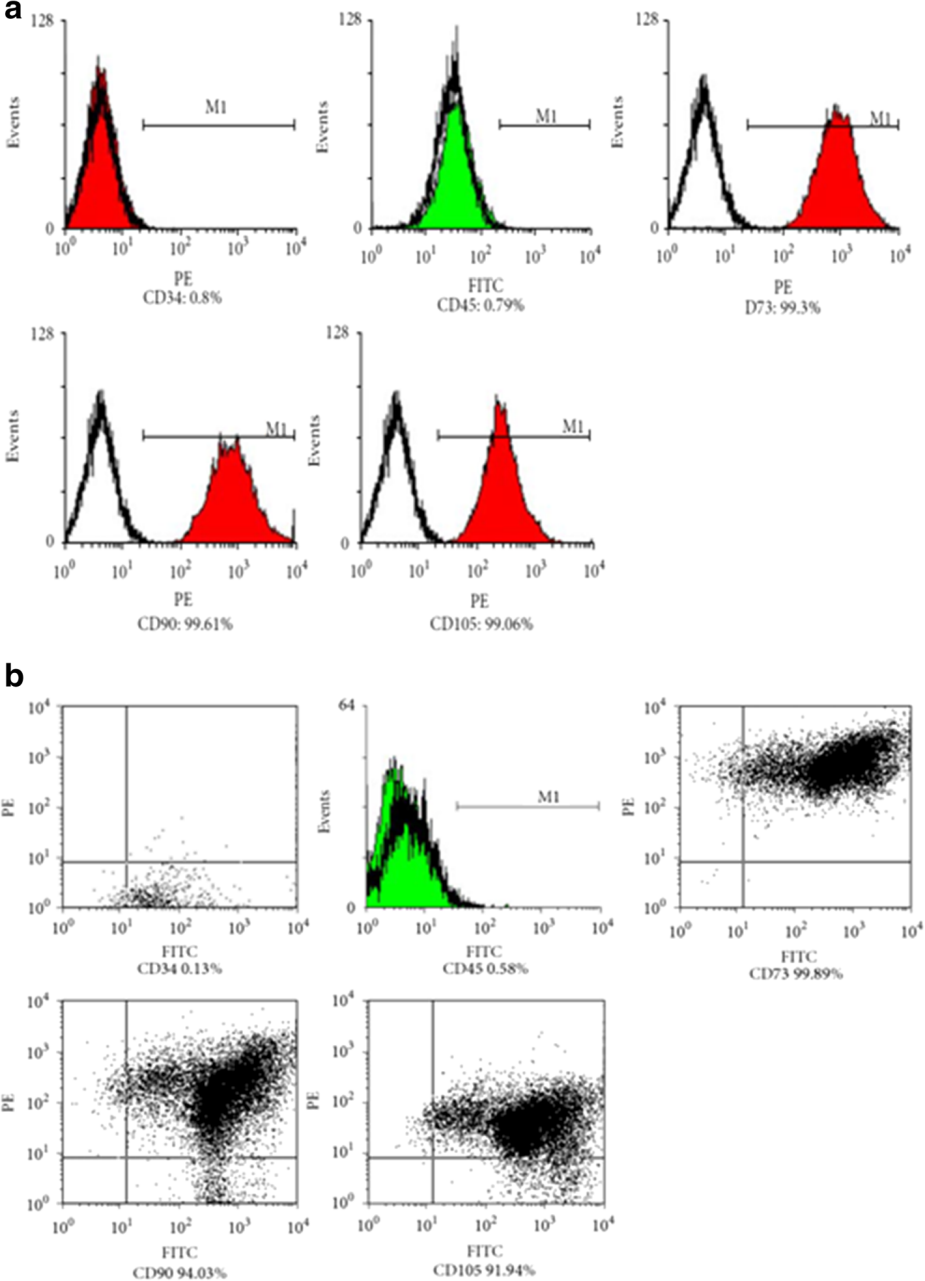
Immunophenotype of WJ-MSCs. Untransfected WJMSCs (**a**) and GFP/SPION-positive WJMSCs (**b**). Flow cytometry data showing negative expression of CD34, CD45 and positive expression of CD73, CD90 and CD105 in the WJMSCs

**Fig. 3:**
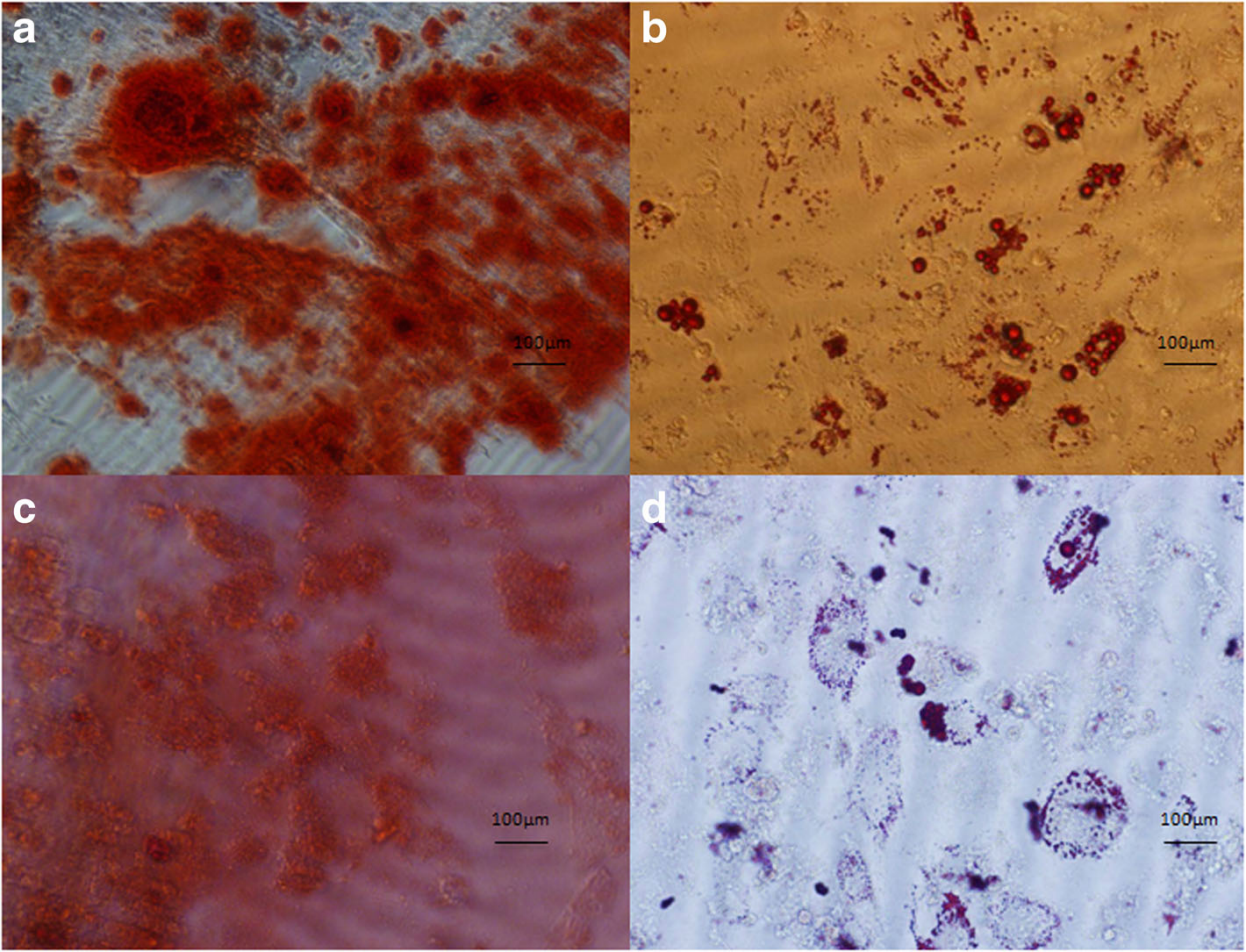
Differentiation of WJMSCs. Osteogenic differentiation analysis of untransfected WJMSCs (**a**) and GFP/SPION-positive WJMSCs (**c**). Adipogenic differentiation analysis of untransfected WJMSCs (**b**) and GFP/SPION-positive WJMSCs (**d**)

The immunophenotype of passage 3 WJ-MSCs, which represent typical fibroblastic cells, and GFP/SPIONs-positive WJ-MSCs was evaluated by flow cytometry. The results showed that all cells expressed CD73, CD105, and CD90 (>95%), but did not express CD34 or CD45 (<2%) (Fig. 2). Furthermore, both untransfected WJ-MSCs and GFP/SPIONs-positive WJ-MSCs were evaluated for their osteogenic and adipogenic differentiation potential. After a 3-week induction under osteogenic conditions, these cells were stained with 0.1% Alizarin Red S water solution. Results showed that majority of the WJ-MSCs were alkaline phosphatase-positive, indicating their osteogenic differentiation potential (Fig. 3a and c). To assess their adipogenic differentiation potential, another culture plate of passage 3 WJ-MSCs was incubated with adipogenic differentiation medium for 3 weeks and then stained with 0.3% Oil Red O. We observed that majority of the cells contained numerous Oil Red O-positive lipid droplets, indicating that WJ-MSCs underwent adipogenic differentiation. (Fig. 3b and d). Growth of GFP/SPIONs-positive WJ-MSCs was seen in the two multiplication cycles; the first multiplication cycle started on day 3 and 4, and the second one in the first 4-7 days. Compared with the control group (HUCMSCs), there was no significant difference at all time points (*t* = 2.05, *p* > 0.05) (Additional file 3: Figure S3).

### In vivo cell tracking using MRI

**Fig. 4:**
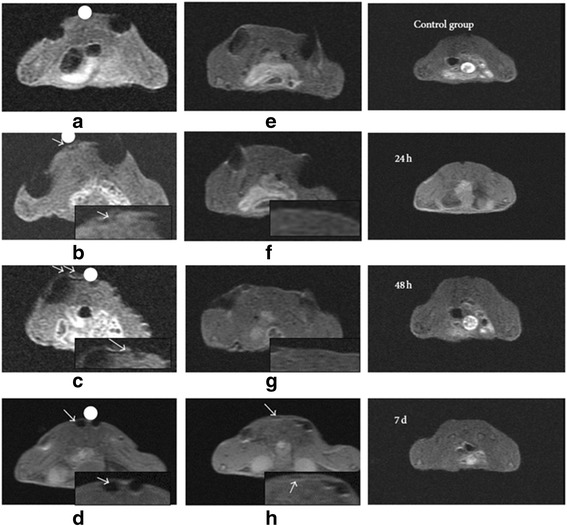
T2-weighted MR images and in vivo cell tracking. Magnetized cells can be detected as hypointense signals (dots): e is FeNdB magnets (8 mm × 2 mm) with a magnetic field of 0.5 T. **a**–**d** the SPIONs-MSCs + magnetic field group, MR images were taken at 0 h (**a**), 24 h (**b**), 48 h (**c**), and 7 d (**d**) after cell transplantation, the MRI image in (**b**) confirms that labeled stem cells (low signal distribution) entered into the trauma center within the first 24 h. **e**–**h** SPIONs-MSCs group without magnetic field, MR images were taken at 0 h (**e**), 24 h (**f**), 48 h (**g**), and 7 d (**h**) after cell transplantation, the 24-h and 48-h MRI images in this group do not demonstrate a low signal area, corresponding to the skin surrounding the wound

**Fig. 5:**
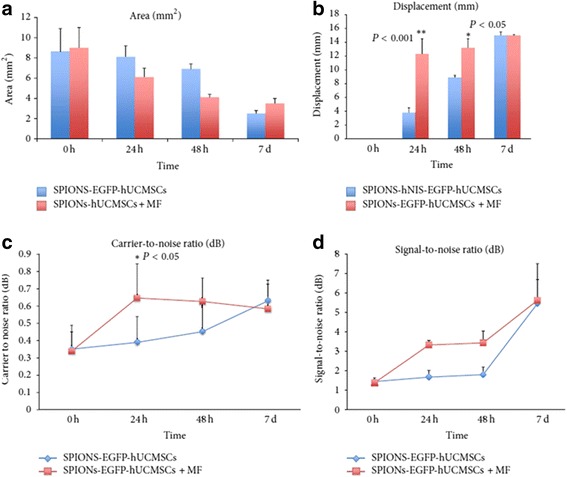
Parameters of MRI image analysis. **a** Reduction in the cluster area of labeled MSCs with time. **b** 24-h (1 d) and 48-h (2 d) displacement of SPIONs-MSCs increased significantly under magnetic field (*P < 0.05, **P < 0.001). **c** CNR was the highest at 24 h, and an MRI shows obvious increase in the SPIONs-MSCs + magnetic field group. **d** SNR increased in the SPIONs-MSCs + magnetic field group by 24 h, and was high in both groups by 7 d

GFP/SPIONs-positive WJ-MSCs were injected around the cutaneous wound areas in injured mice. MRI was performed at 0, 24, and 48 h and 7 days post-transplantation. As expected, GFP/SPIONs-positive WJ-MSCs were successfully directed to the subcutaneous areas of the skin under the influence of the magnetic gradient created by the implanted magnet (Fig. 4). The effect of the EMF on stem cell targeting was significant compared to WJ-MSCs without exposition to magnetic field. The 24-h MR images confirmed that more than 80% of SPIONs/GFP-labeled WJ-MSCs (low signal distribution) reached the trauma center within the first 24 h. However, only low signals were detected around the wounds that were not exposed to the EMF. On day 7, post-cell transplantation, MRI results demonstrated a hypointense signal distribution in the wound center of mice regardless of the magnetic field interference. However, the MRI parameters were not significantly different between the SPIONS-MSCs and SPIONs-MSCs + magnetic field groups. Parameters such as area, SNR, CNR, and displacement can be used as indicators of the concentration and movement speed of cell clusters. In the SPIONs-MSCs + magnetic field group, we observed a significantly reduced area (Fig. 5a) and an increased SNR, CNR, and displacement 24 h, 48 h, and 7 days post-cell transplantation, particularly in the first 24 h (Fig. 5b). The CNR was significantly different between the two groups, especially at the 24 h time point (Fig. 5c). The highest value of SNR was observed at the 7 day time point in the two groups (Fig. 5d).

**Fig. 6:**
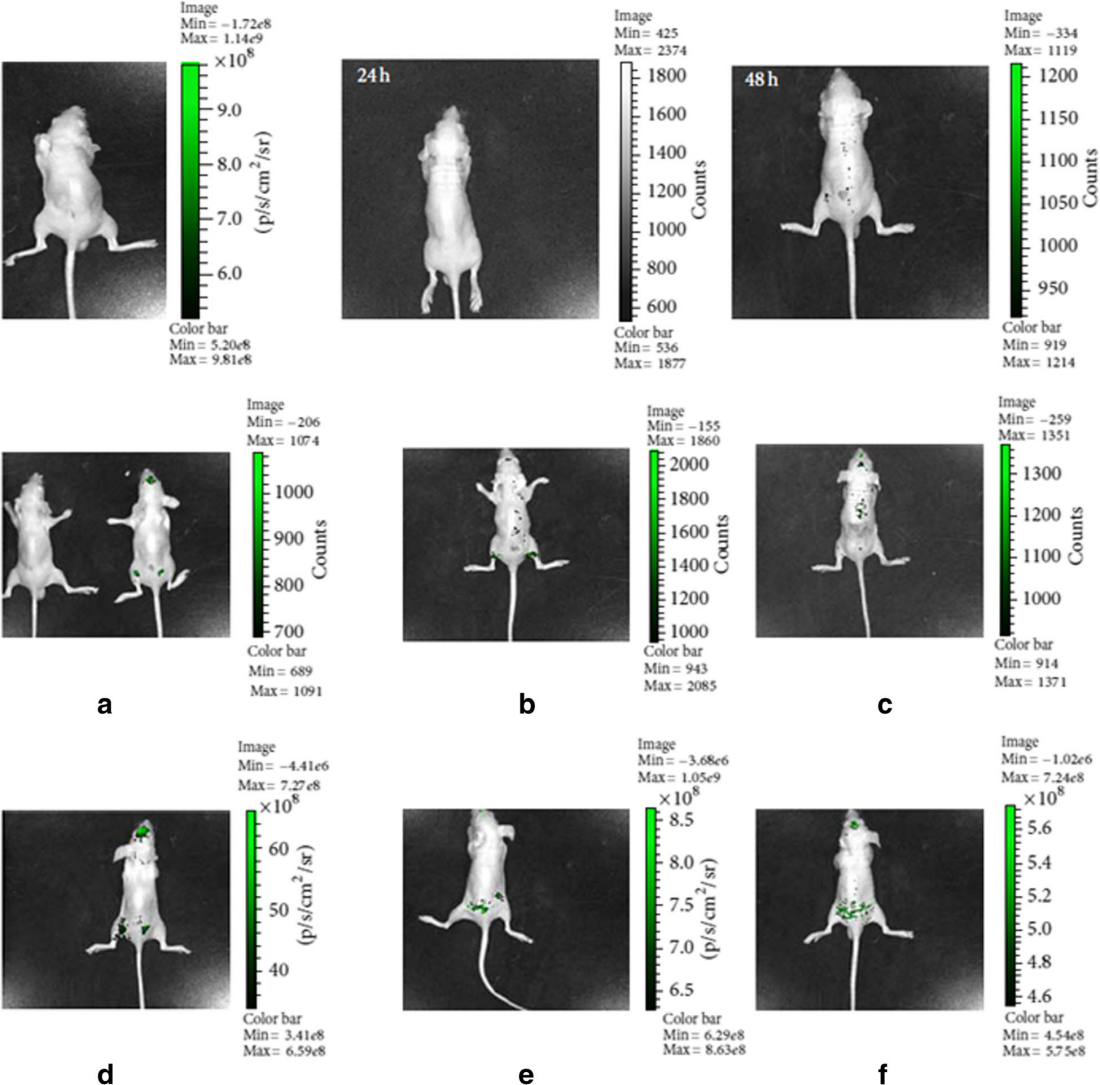
In vivo optical molecular imaging. **d** 0-h, (**e**) 24-h, and (**f**) 48-h images showing the distribution of target cells in the group of SPIONs-MSCs + MF. At 24 h (**e**) and 48 h (**f**), target cell distribution is clear; however, this is not observed in the SPIONs-MSCs group without the MF at the same time points for (**b**) and (**c**) respectively. The (**a**) showes the SPIONs-MSCs group without the MF at 0-h

Moreover, we observed that the wound recovery rate was enhanced in the SPIONs/GFP-MSCs + magnetic field group as compared with the SPIONs/GFP-MSCs group and the control group. Qualitative skin analysis via Prussian blue staining and fluorescence imaging was performed to validate that the hypointense signals detected by MRI indeed corresponded to the presence of MSCs at the target site. The presence of blue cells (Prussian blue) was confirmed in matching skin injury areas, indicating the presence of MSCs in the skin tissue (Fig. 6). Both groups of SPIONs-MSCs, with or without exposure to a magnetic influence, were observed in the skin injury areas of mice 7 days after cell transplantation; however, far fewer positive cells were observed in the SPIONs-MSCs group that did not receive a magnetic implantation. Magnetic implantation, along with injection of SPIONs/GFP-MSCs was found to be safe, as all animals survived and no major signs of tissue injury were observed in vivo by MRI, or ex vivo in the skin tissue.

### In vivo optical molecular imaging

**Fig. 7:**
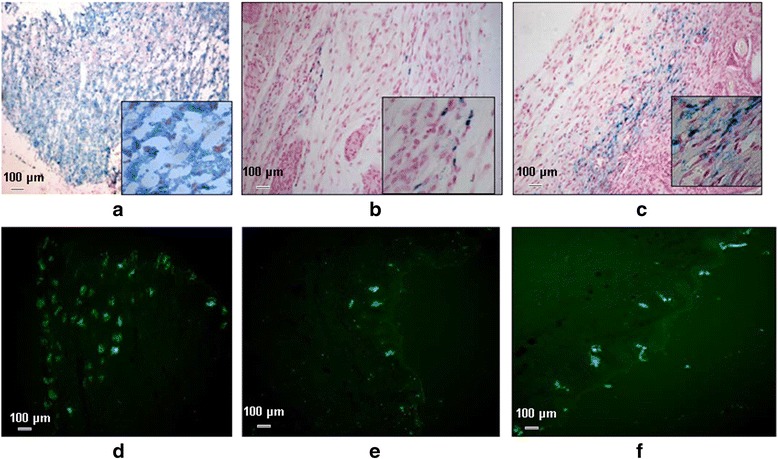
SPIONs and EGFP-labeled human MSCs under magnetic influence identified in skin tissue. **c** and (**f**) show only a few positive cells in the group that was not exposed to the magnetic field. **b** and (**e**) show SPIONs and EGFP-labeled human MSCs after 7 d when the wound healed. **a** and (**d**) show SPIONs and EGFP-labeled human MSCs transplanted subcutaneously in the skin of nude mice at 0 h

To evaluate the role of the magnetic gradient created by the EMF on the activity of SPIONs/GFP-MSCs, we used in vivo optical molecular imaging as a tracer technique to observe the living cells, which express the fluorescent protein. Fluorescence imaging at 0, 24, and 48 h demonstrated a trend of targeted SPIONs-MSCs movement under the EMF, which was consistent with MRI results (Fig. 7). However, the optical molecular imaging technique was not as sensitive as MRI.

## Discussion

Our study demonstrated an EMF-targeted approach that promotes the directional movement of SPION-labeled stem cells, enhancing their ability to repair damaged skin tissues. SPION-labeled human MSCs exhibit excellent paramagnetism, and can accurately target lesion locations under the effect of an EMF, thereby increasing stem cell concentrations at the target site. We confirmed that MSCs doubly labeled with SPIONs and GFP reporter gene demonstrate better dry phenotypes in in vivo experiments. We also demonstrated that SPIONs and GFP labeling does not affect MSC proliferation or vitality, as no significant difference in cell viability was observed before and after labeling. The osteogenic and adipogenic differentiation potential is not affected when the amount of SPIONs-labeled MSCs is 90% [18–21]. In the present study, SPIONs/GFP-labeled MSCs displayed bona fide stem cell features in vitro, similar to that in other studies [22–24]. Weakening of the magnetic flux by exposure to an electromagnetic field, with strength less than 0.1 mT, can promote human umbilical vein endothelial cell proliferation [25]. Exposure of bone marrow-derived MSCs to a 1 mT magnetic field for 1 h per day has been shown to promote cell proliferation and differentiation at an early stage [26–28]. The characteristics of a permanent magnet are far more complex than that of an electromagnetic field, and the force is distance-dependent; therefore, magnetic field properties were not analyzed in this study. There are very few stem cell studies involving the generation of outside target fields by use of a permanent magnet; however, some studies have successfully applied this method. We put an 8 mm × 2 mm permanent magnet with a surface residual magnetism of 0.5 T and a coercive force of 900 KA/m on the surface of the wound, 1.5 cm away from the transplanted cells. The magnetic targeting effect was evident from our results. Since the dead labeled-stem cells also release iron-containing nanoparticles, which can be taken up by the surrounding unlabeled stem cells, we utilized MRI and in vivo fluorescence imaging for synergistic monitoring to avoid any false positive results. However, MRI was more sensitive than in vivo fluorescence imaging. MRI demonstrates high spatial resolution; therefore, a small number of cells can be studied and quantified in a very simple manner using this technique. In the present study, in vivo fluorescence imaging was unable to track cells that had been transplanted for 48 h; however, MRI maintained an excellent tracer capacity until the end of the experiment (up to 7 days). In the case of a 6-h magnetic field exposure each day, both tracer techniques demonstrated that labeled stem cells possess a quick and clear central tendency for magnetic fields. MRI was also very sensitive in demonstrating that a considerable number of stem cells entered the epidermal trauma center within 24 h, and revealed that the maximum displacement of cells extending to the center of the magnetic field was 1.5 cm. Furthermore, using MRI, we also determined that the change in maximum displacement after 48 h was smaller than that in the first 24 h, with a maximum displacement of 0.3 cm. The MRI technique also demonstrated increased cell proliferation in the wound area on day 7 post-cell transplantation. A directed stem cell homing to trauma centers was observed in mice exposed to an EMF; however, other parameters such as displacement, area, and SNR were significantly reduced as compared to in mice that were not exposed to the EMF.

Mice in the EMF group displayed a distinct advantage in the overall wound healing time. The fact that GFP is expressed only in surviving stem cells was used to distinguish the false positive cells, and reflect the true state of surviving stem cells. Its advantage was pronounced in pathological tissue sections, and was confirmed by Prussian blue staining. Fluorescence imaging of pathological tissue sections also demonstrated that the aggregation ability of the targeted stem cells under the effect of an EMF was superior to that of control and non-magnetic field stem cell groups.

MRI tracking of SPION-labeled stem cells has so far proven to be an effective technique, resulting in limited concentration-dependent cellular toxicity. A previous study reported a labeling efficiency of 99%, at concentrations of 25-50 mg Fe/L, without any adverse effects on cell viability, growth, differentiation, and other biological activities [29]. In another study, culture mediums with a SPION concentration of 11.2, 22.4, and 44.8 mg Fe/L led to no changes in stem cell viability and proliferation [30]. Proliferation of liver stem cells has been shown to be inhibited by SPIONs at concentrations higher than 100 mg Fe/L [31]. It has therefore been recommended that a SPION-labeling concentration of 25 mg Fe/L be used [32], which minimally affects the physiological activity of stem cells. In the present study, we used the same SPION-labeling concentration, and observed no changes in cellular morphology or activity.

### Limitations

Our study is limited by the use of relatively few animals, a short duration of the disease, and lack of another control group with only a magnet implant without MSCs injection, to test the effect of magnetic field exposure on skin wound healing. Nevertheless, no histology but the wound area determines whether the skin defect is healed [33]. Moreover, the differentiation potential of SPIONs and GFP-labeled MSCs could have been affected by the iron particles. Further studies are required to explore whether exposure to a magnetic field has the same effect on MSCs from a larger distance and in diseases that are more complex.

## Conclusions

In summary, SPION-labeled stem cells are excellent and safe magnetization and tracer agents, and together with exposure to an EMF generated by permanent magnets, they can be used as a new method of magnetic guidance of targeted stem cells. This method was shown to be safe and effective by MRI and fluorescence analysis of tissue sections. The findings of this study provide a platform for the development of stem cell targeted therapies, and can be further applied for drug and gene targeted therapies.

## Additional files

Additional file 1: Figure S1. The Effect of different external magnetic field exposure time on stem cells was tested by MTT (A), western-blot testing apoptosis marker (B, C). (a) MTT testing showed when the SPION/GFP positive MSCs exposure 6 h/d under magnetic field the cell viability increased, and (b, c) apoptosis marker were low. (TIFF 134 kb)

Additional file 2: Figure S2. Mice were anesthetized with isoflurane (4% induction, 1.5% maintenance), and a small permanent neodymium (FeNdB) magnet (8 × 2 mm) with a magnetic field of 0.5 T was put on the wound of mice for 6 h/day. (TIFF 1394 kb)

Additional file 3: Figure S3. The distributions of cell growth. (TIFF 589 kb)

## Abbreviations

bFGF: Basic fibroblast growth factor
CNR: Carrier-to-noise ratio
EMF: External magnetic field
FBS: Fetal bovine serum
GFP: Green fluorescent protein;
HUCMSCs: Human umbilical cord-derived MSCs
MRI: Magnetic resonance imaging
MSCs: Mesenchymal stem cells
SD: Standard deviation
SNR: Signal-to-noise ratio
SPIONs: Superparamagnetic iron oxide nanoparticles
WJ-MSCs: Wharton’s Jelly of the human umbilical cord-derived MSCs
