# Supplementary figures and images for: External magnetic field promotes homing of magnetized stem cells following subcutaneous injection
Source: BMC Cell Biol. 2017 May 26;18:24. doi: 10.1186/s12860-017-0140-1 (PMC5446710; doi:10.1186/s12860-017-0140-1)

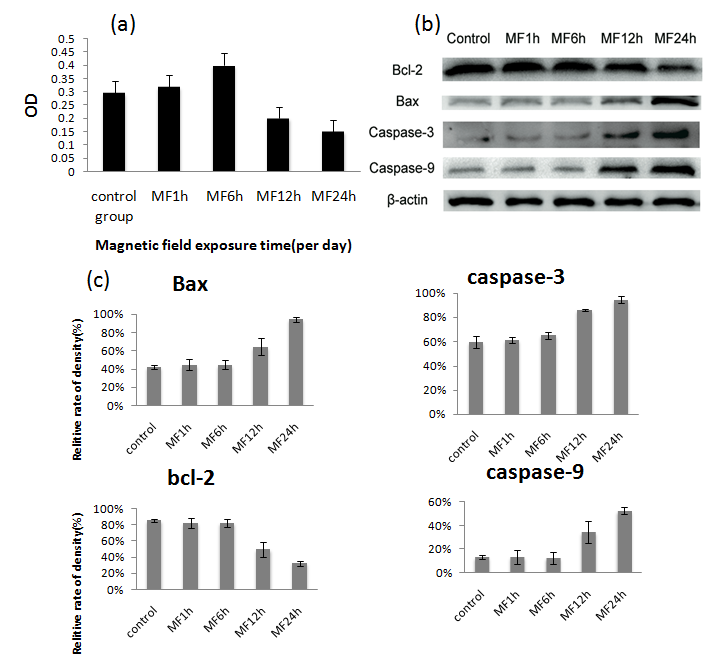

Supplement: Supplementary file 1 — The Effect of different external magnetic field exposure time on stem cells was tested by MTT (A), western-blot testing apoptosis marker (B, C). (a) MTT testing showed when the SPION/GFP positive MSCs exposure 6 h/d under magnetic field the cell viability increased, and (b, c) apoptosis marker were low. (TIFF 134 kb) [file 12860_2017_140_MOESM1_ESM.tif]

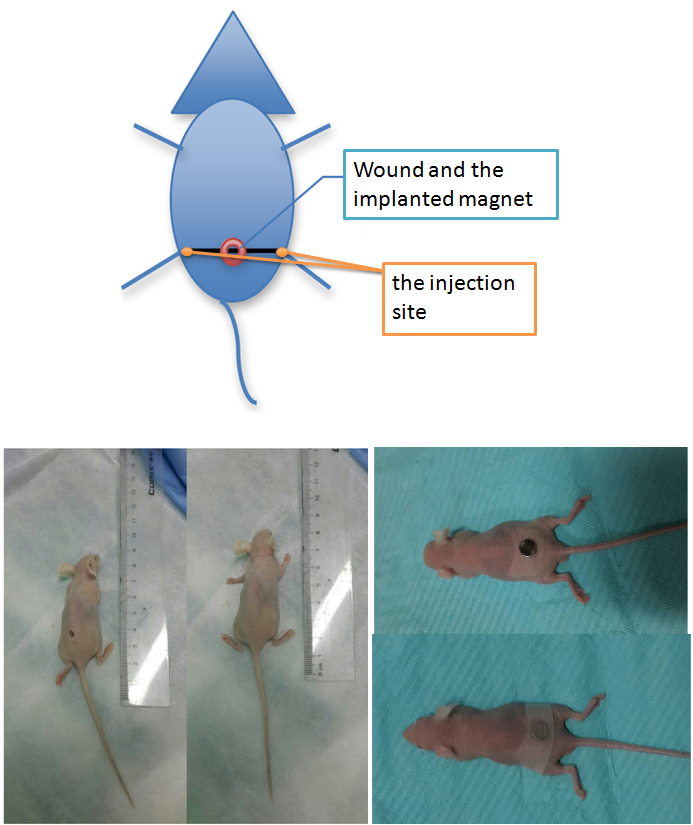

Supplement: Supplementary file 2 — Mice were anesthetized with isoflurane (4% induction, 1.5% maintenance), and a small permanent neodymium (FeNdB) magnet (8 × 2 mm) with a magnetic field of 0.5 T was put on the wound of mice for 6 h/day. (TIFF 1394 kb) [file 12860_2017_140_MOESM2_ESM.tif]

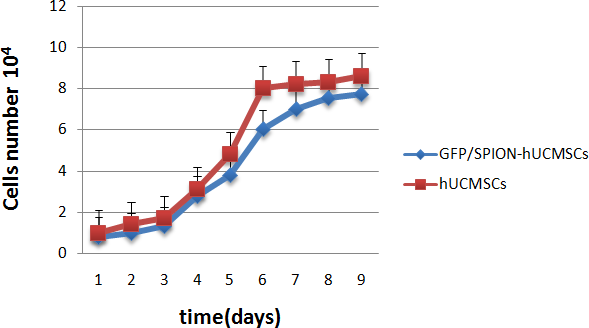

Supplement: Supplementary file 3 — The distributions of cell growth. (TIFF 589 kb) [file 12860_2017_140_MOESM3_ESM.tif]
